# Records of common species of amphibians and reptiles widespread in northern, central, western and southern Ukraine

**DOI:** 10.3897/BDJ.11.e99036

**Published:** 2023-01-31

**Authors:** Oksana Nekrasova, Oleksii Marushchak

**Affiliations:** 1 I. I. Schmalhausen Institute of Zoology of National Academy of Sciences of Ukraine, Kyiv, Ukraine I. I. Schmalhausen Institute of Zoology of National Academy of Sciences of Ukraine Kyiv Ukraine

**Keywords:** Common species, amphibians, reptiles, Ukraine, herpetofauna, GIS-modelling

## Abstract

**Background:**

The dataset includes records of amphibian and reptile species from northern, central, western and southern Ukraine made by Ukrainian herpetologist O. D. Nekrasova during her field trips in the period from 1996 to 2022. Chosen species were not included in the latest published edition of the Red Data Book of Ukraine (2009) and in the latest lists of such species prepared in 2019. The species mentioned in this dataset are characterised by wide range within the country, covering more than 70% of its territory according to spatial distribution modelling (GIS-modelling) made with the help of Maxent software.

**New information:**

The dataset highlights records of eight common species of herpetofauna of Ukraine collected by the first author for the last 26 years. Within the period from 1996 to 2022, O. D. Nekrasova collected and studied information and material on the herpetofauna of the northern, central, western and southern parts of Ukraine from a total of 3960 cadastral points (1707 - for three species of reptiles and 2253 - for five species of amphibians). These records, being now available for the international scientific community, will fill the gap in updated records of the mentioned species, being potentially useful for GIS-modelling, distribution modelling, clarification of conservation lists of national and local importance, further assessment of impact of the war on native biota etc.

## Introduction

The main part of the herpetofauna of Ukraine consists of the so-called "common species", occupying more than 70% of the studied area and numerically prevailing in different biotopes compared to their other classes (Sillero et al. 2014). They tend to be very important parts of many trophic chains in ecosystems, maintaining their energy balance, its flow, keeping a stable state. Simultaneously, the structural and functional state of common species' populations (age composition, sex ratio, morphological and genetic features of these animals) can be used for bioindication, since it reflects the state of the environment as a whole (Yablokov 1987, Akulenko et al. 2019). Amphibian and reptile species, even those treated as common ones, are extremely sensitive to the state of the environment (biotopes and habitats) and, therefore, require development of special conservation measures now, based on the long-term trends and probabilistic models of environmental change (Gibbons et al. 2000, Howard and Bickford 2014, Catenazzi 2015, Greenberg and Palen 2021). Anthropogenic processes demanding such urgent needs include pollution of the environment (e.g. with pecticides, insecticides etc.), degradation, fragmentation and direct destruction of suitable habitats (e.g. as a result of military actions, shelling, using chemical weapons etc.) (Vasyliuk et al. 2022), emergence and expansion of alien invasive species and harmful agents (viruses, microorganisms and parasites) (Kopecký et al. 2013, García‐Díaz et al. 2016, Demkowska-Kutrzepa et al. 2018) and, of course, global climate change (Lindenmayer et al. 2011, Vasyliuk et al. 2015, Tytar et al. 2018, Nekrasova et al. 2019). Common species are usually ecologically flexible and occur both in urbo- and agrocenoses, surviving even in heavily anthropogenically transformed areas (Nekrasova 2002). At the same time, they are characterised by a large number of different morphological and genetic forms and, sometimes in the population, a large number of individuals with anomalies are observed, which have recently increased significantly (Nekrasova 2002, Nekrasova 2008, Nekrasova 2014, Nekrasova and Kuibida 2018, Tytar et al. 2018, Marushchak et al. 2021).

**Figure 1.**
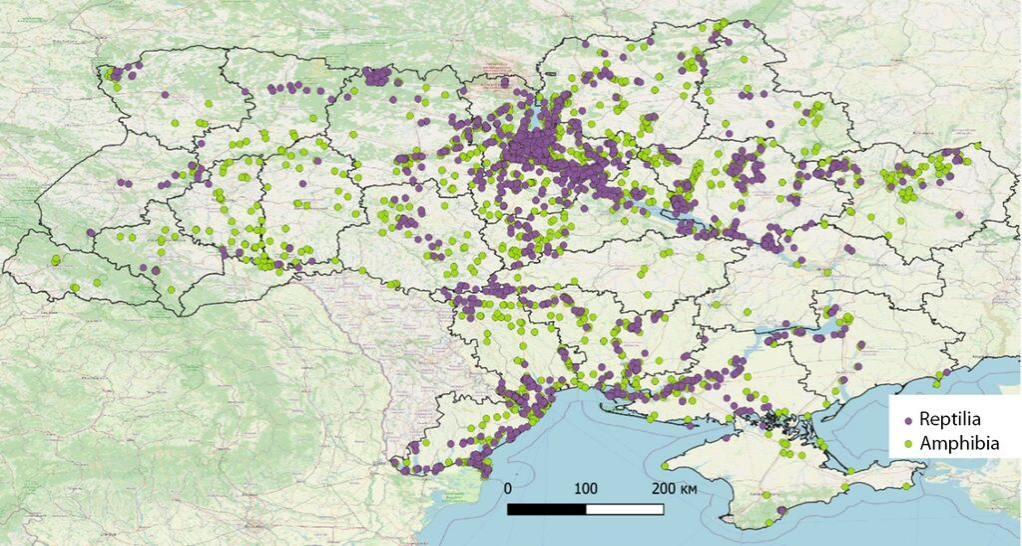
Map of records of studied representatives of herpetofauna (amphians - Amphibia; reptiles - Reptilia) on the territory of Ukraine.

**Figure 2.**
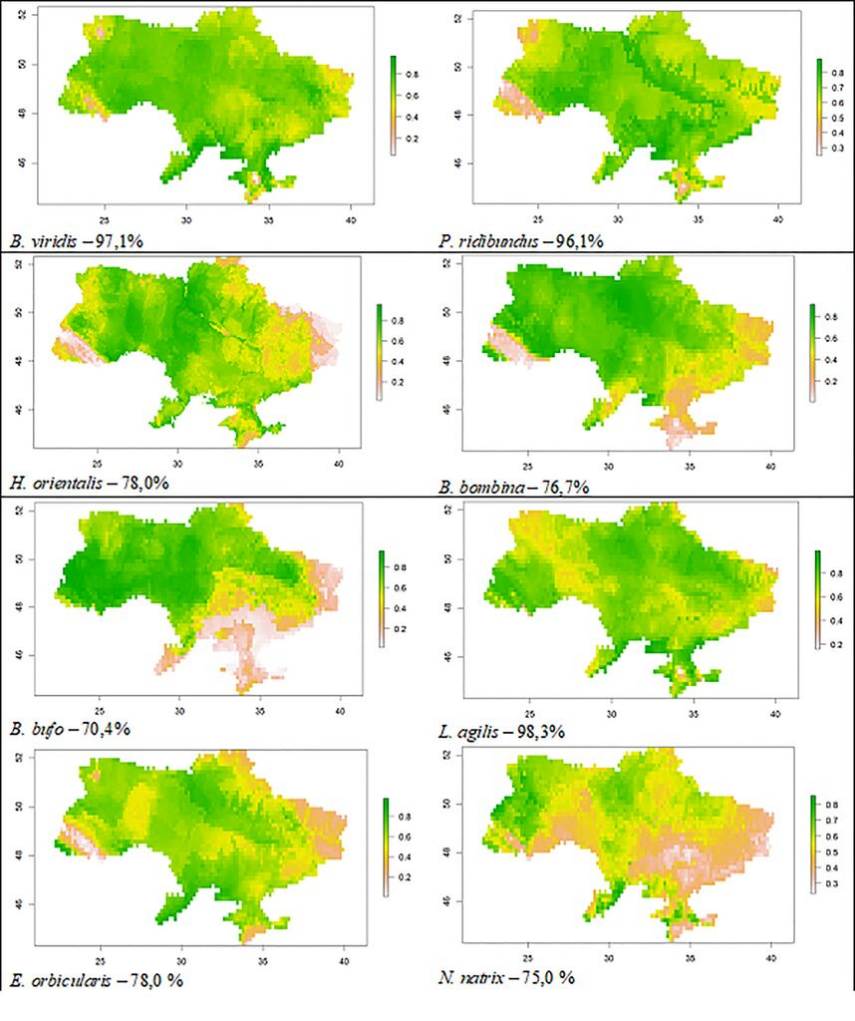
Results of the analysis (Binomial test) of climatically suitable territory of Ukraine (distribution) for the studied amphibian and reptile species.

Amongst the 24 species of reptiles in Ukraine, we have chosen only three "common" species (12.5% of all reptile species inhabiting the country): sand lizard *Lacertaagilis* Linnaeus, 1758; European pond turtle *Emysorbicularis* (Linnaeus, 1758) and grass snake *Natrixnatrix* (Linnaeus, 1758). This group does not include species that are widespread, but are rare and relatively few in numbers or listed in the Red Data Book of Ukraine (Akimov 2009), for example, the smooth snake *Coronellaaustriaca* Laurenti, 1768 (74.1%) (Nekrasova 2014). Amongst 22 species of amphibians in Ukraine, we chose five species of anurans (22.7% of all amphibian species inhabiting Ukraine): semi-aquatic and widespread (https://www.gbif.org/uk/dataset/148bc5c8-0408-424c-84d2-d491ea2e234d) European green toad *Bufotesviridis* (Laurenti, 1768); the most ecologically flexible and numerous marsh frog *Pelophylaxridibundus* (Pallas, 1771); oriental tree frog *Hylaorientalis* (Bedriaga, 1890); European fire-bellied toad *Bombinabombina* (Linnaeus, 1761) widespread in small water bodies and the most vulnerable forest species - common toad *Bufobufo* (Linnaeus, 1758) (Smirnov 2014, Shabanova et al. 2017, Palamarenko 2021) (Table 1, Figs 1, 2).

The published dataset highlights information on widespread amphibian and reptile species, records on the distribution of which in Ukraine has been poorly represented and only available for the scientific community for the last 26 years. The problem is that information on actual records is mainly collected on rare species (listed in national Red Lists of protected species, such as the Red Book of Ukraine) (Akimov 2009). On the contrary, data on common species is usually copied from old publications to the new ones without any real changes (Kuzmin 1999, Pysanets 2007) and, therefore, possible trends, especially negative ones when a common species suddenly becomes rare, can be missed. Tracking and constant updating the evidence on all species (publishing updated bases of records), regardless of their status, is necessary to respond adequately and in a timely manner to their needs and possible population decline. At the very least, the regular publication of such data helps to make adequate changes to the lists of protected species and their status, based on real data (Nekrasova et al. 2019), rather than personal feelings of particular scientists or working groups. Only recently, thanks to the initiatives of the NGO "Ukrainian Nature Conservation Group" on the preparation of the Shadow List of the Emerald Network in Ukraine - newly-proposed territories aimed at the conservation of specific species and habitats mentioned in Resolutions 4 and 6 of the Bern Convention (Vasyliuk et al. 2022) and the initiative of our colleagues to create the National Ukrainian Network UkrBin (UkrBIN 2017) to collect information on the distribution of various species of fauna and flora in Ukraine, this problem started to get solved. Currently, GBIF is one of the most unified international open access databases, which helps to collect and distribute information, including information about common species.

## General description

### Purpose

The dataset consists of records of Amphibia and Reptilia representatives from northern, central, western and southern Ukraine made in the period from 1996 to 2022 (Nekrasova and Marushchak 2022). The species are chosen as those not included in the latest published edition of Red Data Book of Ukraine (Akimov 2009) and in the latest lists of such species prepared in 2019. The species mentioned in this dataset are characterised by wide range within the country, covering more than 70% of its territory according to spatial distribution modelling (GIS-modelling) made with the help of Maxent software. The purpose of the dataset publication is to make the data on common species of herpetofauna of Ukraine available for the scientific community. These data, collected and identified by the first author, are important for tracking of the changes in herpetofauna distribution and population trends in terms of habitat changes induced by climate change, military actions, agriculture intensification and their side effects.

## Sampling methods

### Study extent

Animals were caught manually during the peaks of activity of amphibians or reptiles (mainly from 7:00 to 11:00 am and from 5:00 to 11:00 pm). Both groups of vertebrates were caught mainly by hands. In particular cases for catching amphibians, we used a net or fishing rod, while in cases of catching grass snakes, a hook was used for picking them up. In the vast majority of cases, the animals were simply caught by hand (Pupins et al. 2022). Animals killed on the road by vehicles were taken into account if the identification to specific level was possible. For visualising the records (points) and for further use in GIS-modelling, the points of herpetofauna registrations were collected (with the indication of latitude 00.00000 N and longitude 00.00000 E) using the field off-line orientation programme MAPS.ME (version 12.0.1-Google) and Google Earth Pro (Earth version 7.3.3). Visualisation of records and creation of maps was carried out in the QGIS programme (v.2.181, QGIS Development Team (2016). QGIS Geographic Information System. Open Source Geospatial Foundation. URL http://qgis.org). The species identification was carried out using methodological materials (Bannikov et al. 1977, Nekrasova et al. 2005, Kuzmin 2012).

### Sampling description

The animals were detected using route methods, artificially constructed channels, with the help of special equipment. Animal species were diagnosed by studying larvae and adult individuals and remnants (moulted skin, dead bodies) using morphological methods (Nekrasova 2002, Nekrasova et al. 2005, Nekrasova 2015, Nekrasova et al. 2019), as well as by acoustic data depending on season. Spatial data was used for GIS-analysis, and visualisation of spatial project models and modelling were done using QGIS and DivaGis software (BIOCLIM, DOMAIN algorithms). To account for potential sampling bias, we used the nearest-neighbour distance ('ntbox' package in R) method (Osorio‐Olvera et al. 2020) to thin the data: to avoid spatial autocorrelation, occurrence points ≤ 0.1 units (meaning approximately the spatial resolution of the climate factors’ database (.tiff map file, 2.5’ (or approximately 5 km) spatial resolution) used for the research) away from each other were removed. We used 19 bioclimatic indicators (Table 2) from the database from the CliMond dataset (Kriticos et al. 2014) https://www.climond.org/ (accessed 27 December 2020), A1B scenario). Species distribution modelling (SDM) methods (Anderson et al. 2003, Araújo et al. 2019) have been employed to explore the potential climatically suitable territories for the studied species within the territory of Ukraine. Binomial test was used for building distribution maps when choosing the species for study. It helps to make a significance estimation of a niche model by using the cumulative binomial probability of success of predicting an occurrence given the validation data and the proportional area predicted as present in the niche model (Anderson et al. 2003). The modelling was done in the Maxent v.3.4.4 software (Phillips et al. 2006, Peterson et al. 2008) with default settings. This software was chosen as Maxent, which, unlike other distributional modelling techniques, uses only presence and background data instead of presence and absence data. Visualisation of the models was carried out using programmes - SAGA GIS and QGis (Nekrasova et al. 2019).

### Quality control

Both authors are professional herpetologists specialising in, amongst other topics, Ukrainian herpetofauna, which is proved by a great number of relevant publications. Authors of the dataset are fully responsible for the quality of data provided in it: georeferenced locations, species identification, time when record was made etc.

### Step description

1. Conducting of field surveys and trips in search for representatives of native herpetofauna.
2. Collecting of information from other local residents.
3. Visual species identification as well as number of individuals.
4. Georeferencing.
5. Taking photos and necessary measurements, if needed.
6. Putting of the information in the digital table designed in MS Excel.
7. Organising of a dataset according to DarwinCore standards.

## Geographic coverage

### Description

The dataset covers the entire territory of Ukraine. It hightlights records of representatives of native herpetofauna within all regions of Ukraine, except Luhansk and Donetsk Regions.

### Coordinates

44.402 and 52.376 Latitude; 22.192 and 40.122 Longitude.

## Taxonomic coverage

### Description

The dataset consists of records of the most common species of reptiles and amphibians inhabiting the territory of Ukraine, namely, representatives belonging to Hylidae, Bombinatoridae, Bufonidae, Ranidae, Colubridae, Lacertidae and Emydidae families.

### Taxa included

**Table:** 

Rank	Scientific Name	
kingdom	Animalia	
phylum	Chordata	
class	Amphibia	
order	Anura	
family	Bombinatoridae	
family	Bufonidae	
family	Hylidae	
family	Ranidae	
class	Reptilia	
order	Squamata	
family	Colubridae	
family	Lacertidae	
order	Testudines	
family	Emydidae	

## Temporal coverage

**Formation period:** 1996/2022.

## Usage licence

### Usage licence

Open Data Commons Attribution License

## Data resources

### Data package title

Records of common herpetofauna species widespread in northern, central, western and southern Ukraine

### Resource link

https://www.gbif.org/uk/dataset/148bc5c8-0408-424c-84d2-d491ea2e234d

### Alternative identifiers

https://doi.org/10.15468/3t8srm

### Number of data sets

1

### Data set 1.

#### Data set name

Records of common species of amphibians and reptiles widespread in northern, central, western and southern Ukraine

#### Data format

Darwin Core

#### Data format version

1.9

#### Description

In the "occurrenceRemarks" column of the dataset, "REL-population" means a mixed population of green frogs (genus *Pelophylax*) consisting of the three known species for Ukraine, namely *P.ridibundus*, *Pelophylaxlessonae* (Camerano, 1882) and *Pelophylaxesculentus* (Linnaeus, 1758). "RE-population" means the mixed population as well, but consisting of *P.ridibundus* and *P.esculentus* individuals only.

**Data set 1.** 

Column label	Column description
occurrenceID	http://rs.tdwg.org/dwc/terms/occurrenceID; a unique identifier of a particular occurrence within this dataset.
scientificName	http://rs.tdwg.org/dwc/terms/scientificName; the full scientific latin name of the recorded species.
basisOfRecord	http://rs.tdwg.org/dwc/terms/basisOfRecord; the specific type of the record, based mainly on the way in which it was made.
eventDate	http://rs.tdwg.org/dwc/terms/eventDate; the exact date or interval during which the record was made.
verbatimeventDate	http://rs.tdwg.org/dwc/terms/verbatimEventDate; the verbatim original representation of the date information for the specific record.
year	http://rs.tdwg.org/dwc/terms/year; the year in which the event occurred.
occurrenceRemarks	http://rs.tdwg.org/dwc/terms/occurrenceRemarks; comments or notes about the occurrence.
organismQuantity	http://rs.tdwg.org/dwc/terms/organismQuantity; a number or enumeration value for the quantity of the recorded organisms.
organismQuantityType	http://rs.tdwg.org/dwc/terms/organismQuantityType; the type of quantification system used for the quantity of organisms.
taxonRank	http://rs.tdwg.org/dwc/terms/taxonRank; the taxonomic rank of the most specific name in the scientificName as it appears in the original record.
decimalLatitude	http://rs.tdwg.org/dwc/terms/decimalLatitude; the geographic latitude (in decimal degrees, using the spatial reference system given in geodeticDatum) of the geographic centre of a location, where the record was made.
decimalLongitude	http://rs.tdwg.org/dwc/terms/decimalLongitude;the geographic longitude (in decimal degrees, using the spatial reference system given in geodeticDatum) of the geographic centre of a location, where the record was made.
language	http://purl.org/dc/terms/language; a language of the resource.
identifiedBy	http://rs.tdwg.org/dwc/iri/identifiedBy; a person who assigned the taxon to the subject.
identifiedbyID	http://rs.tdwg.org/dwc/terms/identifiedByID;a globally unique identifier for the person responsible for assigning the taxon to the subject (in this dataset, this is an ORCID record).
geodeticDatum	http://rs.tdwg.org/dwc/iri/geodeticDatum;the geodetic datum or spatial reference system (SRS) upon which the geographic coordinates given in decimalLatitude and decimalLongitude are based (WGS84).
georeferencedBy	http://rs.tdwg.org/dwc/terms/georeferencedBy; a person or group of people who determined the georeference (spatial representation) for the location.
georeferenceProtocol	http://rs.tdwg.org/dwc/terms/georeferenceProtocol; a short description or reference to the methods used to determine the spatial footprint, coordinates and uncertainties.
coordinatesUncertaintyInMetres	http://rs.tdwg.org/dwc/terms/coordinateUncertaintyInMeters;the horizontal distance (in metres) from the given decimalLatitude and decimalLongitude describing the smallest circle containing the whole of the location area.
continent	http://rs.tdwg.org/dwc/terms/continent; the name of the continent in which the location occurs.
countryCode	http://rs.tdwg.org/dwc/terms/countryCode; the standard code for the country in which the location occurs.
country	http://rs.tdwg.org/dwc/terms/country; the name of the country or major administrative unit in which the location occurs.
stateProvince	http://rs.tdwg.org/dwc/terms/stateProvince; the name of the next smaller administrative region than country (oblast' or region) in which the location occurs.
kingdom	http://rs.tdwg.org/dwc/terms/kingdom;the full scientific name of the kingdom in which the taxon is classified.
phylum	http://rs.tdwg.org/dwc/terms/phylum;the full scientific name of the phylum in which the taxon is classified.
class	http://rs.tdwg.org/dwc/terms/class;the full scientific name of the class in which the taxon is classified.
order	http://rs.tdwg.org/dwc/terms/order;the full scientific name of the order in which the taxon is classified.
family	http://rs.tdwg.org/dwc/terms/family;the full scientific name of the family in which the taxon is classified.
genus	http://rs.tdwg.org/dwc/terms/genus;the full scientific name of the genus in which the taxon is classified.
specificEpithet	http://rs.tdwg.org/dwc/terms/specificEpithet;the name of the first or species epithet of the scientificName.
recordedBy	http://rs.tdwg.org/dwc/terms/recordedBy;a person or several people, who made the record.
recordedByID	ttp://rs.tdwg.org/dwc/terms/recordedByID; a globally unique identifier for the person, responsible for recording the original occurrence.
type	http://purl.org/dc/elements/1.1/type; the nature or genre of the resource.

## Figures and Tables

**Table 1.** A list of the studied amphibians and reptiles of Ukrainian herpetofauna.

AMPHIBIA			Conservation status *	N	% of the territory of Ukraine
Number	Family	Species			
1.	Bombinatoridae	*Bombinabombina* (Linnaeus, 1761)	IUCN (LС), BC (2)	532	76.7
2.	Bufonidae	*Bufobufo* (Linnaeus, 1758)	IUCN (LС), BC (3)	210	70.4
3.	Bufonidae	*Bufoviridis* Laurenti, 1768	IUCN (LС), BC (2)	354	97.1
4.	Hylidae	*Hylaorientalis* (Bedriaga, 1890)	IUCN (LС), BC (2)	312	78.0
5.	Ranidae	*Pelophylaxridibundus* (Pallas, 1771)	IUCN (LС), BC (3)	845	96.1
			Total:	2253	
REPTILIA					
6.	Emydidae	*Emysorbicularis* (Linnaeus,1758)	IUCN (NT), BC (2)	583	78.0
7.	Lacertidae	*Lacertaagilis* Linnaeus, 1758	IUCN (LС), BC (2)	615	98.3
8.	Colubridae	*Natrixnatrix* (Linnaeus, 1758)	IUCN (LR/LC), BC (3)	509	75.0
			Total:	1707	

**Table 2.** Bioclimatic variables (19) from the CliMond dataset (Kriticos et al. 2014; https://www.climond.org/).

Variable	Meaning
bio1	Annual mean temperature (°C)
bio2	Mean diurnal temperature range (mean (period max-min)) (°C)
bio3	Isothermality (bio02 ÷ bio07)
bio4	Temperature seasonality (C of V)
bio5	Max temperature of warmest week (°C)
bio6	Min temperature of coldest week (°C)
bio7	Temperature annual range (bio05-bio06) (°C)
bio8	Mean temperature of wettest quarter (°C)
bio9	Mean temperature of driest quarter (°C)
bio10	Mean temperature of warmest quarter (°C)
bio11	Mean temperature of coldest quarter (°C)
bio12	Annual precipitation (mm)
bio13	Precipitation of wettest week (mm)
bio14	Precipitation of driest week (mm)
bio15	Precipitation seasonality (C of V)
bio16	Precipitation of wettest quarter (mm)
bio17	Precipitation of driest quarter (mm)
bio18	Precipitation of warmest quarter (mm)
bio19	Precipitation of coldest quarter (mm)
